# From a long-distance threat to the invasion front: a review of the invasive *Aedes* mosquito species in Belgium between 2007 and 2020

**DOI:** 10.1186/s13071-022-05303-w

**Published:** 2022-06-13

**Authors:** Isra Deblauwe, Katrien De Wolf, Jacobus De Witte, Anna Schneider, Ingrid Verlé, Adwine Vanslembrouck, Nathalie Smitz, Julie Demeulemeester, Thomas Van Loo, Wouter Dekoninck, Meryam Krit, Maxime Madder, Ruth Müller, Wim Van Bortel

**Affiliations:** 1 The Unit of Entomology, Department Biomedical Sciences, Institute of Tropical Medicine, Antwerp, Belgium; 2 Terrestrial Ecology Unit, Department of Biology, Ghent University, Ghent, Belgium; 3 Royal Museum for Central Africa (BopCo), Tervuren, Belgium; 4 Inagro Vzw, Rumbeke-Beitem, Belgium; 5 Royal Belgian Institute of Natural Sciences (Scientific Heritage Service), Brussels, Belgium; 6 The Unit of Eco-Modelling, Department Biomedical Sciences, Institute of Tropical Medicine, Antwerp, Belgium; 7 Clinglobal, Tamarin, Mauritius; 8 Department of Veterinary Tropical Diseases, University of Pretoria, Onderstepoort, South Africa; 9 Outbreak Research Team, Institute of Tropical Medicine, Antwerp, Belgium

**Keywords:** *Aedes albopictus*, *Aedes japonicus japonicus*, *Aedes koreicus*, Mosquito monitoring, Exotic mosquito species, Surveillance, Introduction pathways, Establishment, Point of entry, Culicidae

## Abstract

Invasive mosquito species (IMS) and their associated mosquito-borne diseases are emerging in Europe. In Belgium, the first detection of *Aedes albopictus* (Skuse 1894) occurred in 2000 and of *Aedes japonicus japonicus* (Theobald 1901) in 2002. Early detection and control of these IMS at points of entry (PoEs) are of paramount importance to slow down any possible establishment. This article reviews the introductions and establishments recorded of three IMS in Belgium based on published (2007–2014) and unpublished (2015–2020) data collected during several surveillance projects. In total, 52 PoEs were monitored at least once for the presence of IMS between 2007 and 2020. These included used tyre and lucky bamboo import companies, airports, ports, parking lots along highways, shelters for imported cutting plants, wholesale markets, industrial areas, recycling areas, cemeteries and an allotment garden at the country border with colonised areas. In general, monitoring was performed between April and November. Mosquitoes were captured with adult and oviposition traps as well as by larval sampling. *Aedes albopictus* was detected at ten PoEs, *Ae. japonicus* at three PoEs and *Aedes koreicus* (Edwards 1917) at two PoEs. The latter two species have established overwintering populations. The percentage of PoEs positive for *Ae. albopictus* increased significantly over years. *Aedes albopictus* is currently entering Belgium through lucky bamboo and used tyre trade and passive ground transport, while *Ae. japonicus* through used tyre trade and probably passive ground transport. In Belgium, the import through passive ground transport was first recorded in 2018 and its importance seems to be growing. Belgium is currently at the invasion front of *Ae. albopictus* and *Ae. japonicus*. The surveillance and control management actions at well-known PoEs associated to long-distance introductions are more straightforward than at less-defined PoEs associated with short-distance introductions from colonised areas. These latter PoEs represent a new challenge for IMS management in Belgium in the coming years. *Aedes albopictus* is expected to become established in Belgium in the coming years, hence increasing the likelihood of local arbovirus transmission. The implementation of a sustainable, structured and long-term IMS management programme, integrating active and passive entomological surveillance, vector control and Public Health surveillance is therefore pivotal.

**Graphical Abstract:**

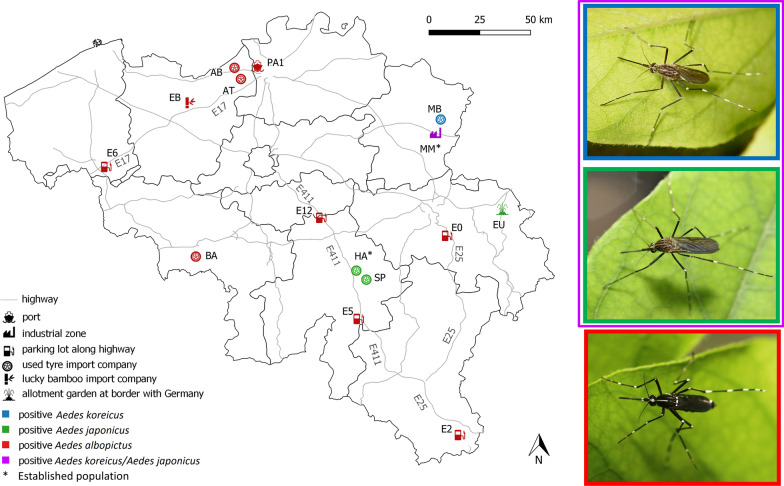

**Supplementary Information:**

The online version contains supplementary material available at 10.1186/s13071-022-05303-w.

## Background

The invasive mosquito species (IMS) *Aedes aegypti* (Linnaeus, 1762), *Aedes albopictus* (Skuse 1894), *Aedes japonicus japonicus* (Theobald 1901) and *Aedes koreicus* (Edwards 1917) have been proven to be vectors of several arboviruses causing mosquito-borne diseases [1, 2]. These IMS adapted to the human environment by using artificial containers as larval habitat (such as tyres, rain water barrels and catch basins), which contributed to their invasive range expansion [3]. The increased international movement of goods and people, together with climate warming and urbanisation, have ensured the global expansion of IMS and is expected to continue in the future [4–8]. Autochthonous outbreaks of mosquito-borne diseases typically follow 5–15 years after the establishment of *Aedes albopictus* [6]. Mosquito-borne diseases are a growing threat for public and animal health in Europe [9, 10]. Local transmission events of dengue (DENV), chikungunya (CHIKV) and Zika (ZIKV) viruses occur in Europe, primarily in Mediterranean countries [11–13]. A prerequisite for autochthonous disease transmission is the introduction of pathogens and the presence of a competent vector. The World Health Organisation (WHO) stressed the significance of building capacities to detect, assess and report public health events with capacity building for vector surveillance and control at PoEs as one of the essential elements of the WHO International Health Regulations [14, 15]. This would aid the early detection and control of IMS, which is of paramount importance to slow down any possible establishment of IMS in a given area.

*Aedes albopictus* and *Ae. japonicus* are IMS species that have established in multiple countries in Europe over the last 2 decades. Following the first observations of *Ae. albopictus* and *Ae. japonicus* in France in 1999 [16] and 2000 [17], respectively, three Belgian companies that import used tyres and export to France were inspected between 2000 and 2003. The Belgian companies were targeted because of possible import of tyres from countries where *Ae. albopictus* is native or established [18]. In 2000, *Ae. albopictus* was detected for the first time in Belgium on the premises of a used tyre import company in Vrasene [19], but did not overwinter. The first collection of *Ae. japonicus* in Belgium was at a used tyre import company in Natoye in 2002, where it was detected again in 2003 and 2004, but did not spread to the surroundings at that time [18]. Despite these early detections, IMS surveillance was initiated in Belgium not earlier than 2007. This article reviews the introductions and establishments recorded of three IMS in Belgium based on published (2007–2014) and unpublished (2015–2020) data collected during several surveillance projects.

## Overview of the project-based surveillance methodology

### Surveillance projects

Since 2007, several projects have been completed to survey IMS in Belgium: MODIRISK [20–23], EXOSURV [24, 25], FASCF [26], MEMO [27] and MEMO+2020 [28]. First, the large-scale national inventory study (MODIRISK, 2007–2010) aimed to advance our restricted knowledge on mosquito biodiversity and distribution in Belgium [20, 23]. Second, the pilot project EXOSURV (2012) evaluated the usefulness and applicability of the ECDC guidelines for IMS surveillance [29] by setting up IMS-focused surveillance for the first time in Belgium [24]. Third, exotic vectors and pathogens were surveyed between 2013 and 2016 (FASFC project) [26]. Fourth, a national monitoring of exotic mosquito species was conducted between 2017 and 2020 (MEMO [27] and MEMO+2020 [28] projects), complemented in 2020 by the DiMoC project on diversity components in mosquito-borne diseases in the face of climate change. A detailed overview of the IMS surveillance projects with their sampling strategies tailored to specific entomological risk scenarios (see also ‘Sampling strategies’) is presented in Additional file 1: Table S1.

### Selection of the points of entry (PoEs)

**Fig. 1:**
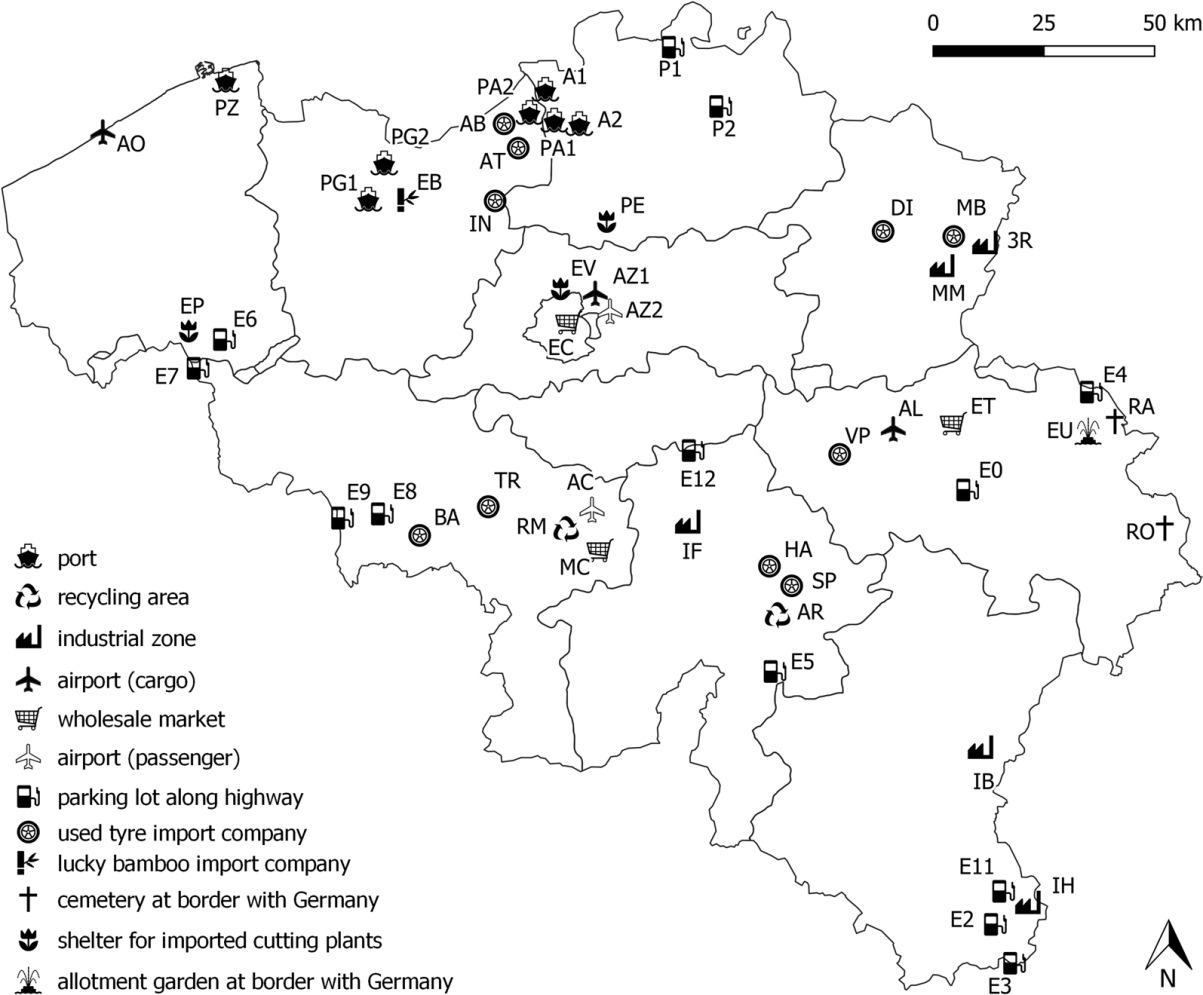
Map of Belgium with 52 monitored PoEs for invasive mosquito species (IMS). The borders indicate the areas of Belgian provinces

The following known and potential import routes and locations near country borders (referred to as ‘points of entry’ or PoEs) were monitored in Belgium: used tyre and lucky bamboo import companies, airports, ports, parking lots along highways, shelters for imported cutting plants, wholesale markets, industrial areas, recycling areas, and cemeteries as well as an allotment garden at the country border with colonised areas. In total 52 PoEs were monitored at least once for IMS between 2007 and 2020 (Fig. 1, Table 1).

**Table 1:** Overview of the 52 points of entry (PoEs) monitored in Belgium between 2007 and 2020, indicating the PoE type, municipality, geographical coordinates, risk scenario per year and invasive mosquito species (IMS) detected and number of years monitored

PoE code	PoE type	Municipality	Latitude	Longitude	MODIRISK				EXOSURV	FASFC				MEMO			MEMO +2020/DiMoC	IMS detected	Total no. years monitored
					2007	2008	2009	2010	2012	2013	2014	2015	2016	2017	2018	2019	2020		
AL	Airport (cargo)	Grâce-Hollogne	50.637071	5.430389					SC1	SC1	SC1	SC1		SC1	SC1	SC1			7
AZ1	Airport (cargo)	Zaventem	50.906050	4.463900	SC1				SC1	SC1	SC1	SC1		SC1					6
AO	Airport (cargo)	Ostend	51.204808	2.874425	SC1				SC1	SC1	SC1	SC1							5
AC	Airport (passenger)	Charleroi	50.469697	4.471293										SC1	SC1	SC1			3
AZ2	Airport (passenger)	Zaventem	50.899057	4.483719											SC1	SC1			2
EU	Allotment garden at border with Germany	Eupen	50.632399	6.073232										(SC1)^a^	SC1 INS	SC1 INS		*Ae. japonicus*	3
RO	Cemetery at border with Germany	Büllingen	50.432761	6.294764										SC1	SC1	SC1			3
RA	Cemetery at border with Germany	Raeren	50.675553	6.110327										SC1					1
MM	Industrial zone	Maasmechelen	50.995255	5.621248		SC2	SC2		SC2	SC2	SC2	SC2		SC2	SC1/SC3	SC3^b^		*Ae. koreicus, Ae. japonicus*	9
IB	Industrial zone	Bastogne	49.988369	5.705020		SC1													1
IH	Industrial zone	Arlon	49.648972	5.828590		SC1													1
IF	Industrial zone	Floreffe	50.445179	4.776840		SC1													1
3R	Industrial zone	Dilsen-Stokkem	51.014159	5.697125					SC1										1
EB	Lucky bamboo import company	Lochristi	51.112279	3.836114	SC1	SC1			SC1	SC1	SC1 INS	SC1 INS	SC1 INS	SC1	SC1 INS	SC1 INS		*Ae. albopictus*	10
E7	Parking lot along highway	Rekkem	50.764356	3.172908					SC1	SC1	SC1	SC1		SC1					5
E2	Parking lot along highway	Messancy	49.643818	5.830286					SC1					SC1	SC1 INS	SC1		*Ae. albopictus*	4
E4	Parking lot along highway	Raeren	50.717536	6.117821										SC1	SC1	SC1	SC1		4
E6	Parking lot along highway	Kortrijk	50.799111	3.252428											SC1	SC1 INS	SC1	*Ae. albopictus*	3
E8	Parking lot along highway	Saint-Ghislain	50.451261	3.804774											SC1	SC1	SC1		3
E12	Parking lot along highway	Eghezée	50.600423	4.792036												SC1	SC1 INS	*Ae. albopictus*	2
E3	Parking lot along highway	Aubange	49.550703	5.808331					SC1					SC1					2
E5	Parking lot along highway	Houyet	50.144183	5.079253											SC1 INS	SC1 INS		*Ae. albopictus*	2
E0	Parking lot along highway	Sprimont	50.524368	5.667709												SC1 INS		*Ae. albopictus*	1
E11	Parking lot along highway	Arlon	49.676605	5.784327					SC1										1
E9	Parking lot along highway	Hensies	50.438789	3.670263										SC1					1
P1	Parking lot along highway	Hoogstraten	51.419962	4.707289													SC1		1
P2	Parking lot along highway	Vosselaar	51.291587	4.872080													SC1		1
PG1	Port	Ghent	51.108754	3.751126	SC1				SC1	SC1	SC1	SC1							5
A1	Port	Antwerp	51.329414	4.345138	SC1				SC1										2
A2	Port	Antwerp	51.252230	4.395134	SC1				SC1										2
PG2	Port	Ghent	51.159808	3.780577											SC1^c^				1
PZ	Port	Zeebrugge	51.337907	3.203598										SC1					1
PA2	Port (left bank)	Kallo	51.278834	4.276499										SC1	SC1^c^				2
PA1	Port (right bank)	Antwerp	51.263685	4.360237						SC1	SC1	SC1						*Ae. albopictus*	3
AR	Recycling area	Achêne	50.260330	5.063560		SC1													1
RM	Recycling area	Monceau-sur-Sambre	50.431431	4.389880		SC1													1
EV	Shelter for imported cutting plants	Brussels	50.888243	4.382161					SC1	SC1	SC1	SC1				SC1			5
EP	Shelter for imported cutting plants	Wevelgem	50.815885	3.211485					SC1										1
PE	Shelter for imported cutting plants	Sint-Katelijne-Waver	51.055363	4.507212					SC1										1
AT	Used tyre import company	Vrasene	51.213511	4.193505	SC1	SC1	SC1		SC1	SC1 INS	SC1	SC1	SC1 INS	SC1	SC1	SC1		*Ae. albopictus*	11
MB	Used tyre import company	Dilsen-Stokkem	51.016332	5.695365		SC1		SC1			SC1	SC1		SC1	SC1	SC1		*Ae. koreicus*	7
VP	Used tyre import company	Villers-Le-Bouillet	50.585328	5.259748					SC1	SC1	SC1	SC1		SC1	SC1	SC1			7
HA	Used tyre import company	Natoye	50.338961	5.045369	SC2		SC2	SC2	SC2					SC2	SC2^b^	SC2^b^	SC2^b^	*Ae. japonicus*	8
BA	Used tyre import company	Frameries	50.412026	3.924913									SC1 INS	SC1	SC1 INS	SC1		*Ae. albopictus*	4
SP	Used tyre import company	Natoye	50.335863	5.071636		SC2			SC2^b^					SC1	SC1			*Ae. japonicus*	4
AB	Used tyre import company	Kallo	51.251563	4.217950										SC1	SC1 INS	SC1		*Ae. albopictus*	3
DI	Used tyre import company	Houthalen-Helchteren	51.038874	5.398481		SC1	SC1		SC1										3
TR	Used tyre import company	La Louvière	50.472498	4.141277					SC1	SC1									2
IN	Used tyre import company	Hamme	51.094059	4.146750		SC1													1
EC	Wholesale market	Brussels	50.878371	4.371272					SC1	SC1	SC1	SC1							4
ET	Wholesale market	Liège	50.648771	5.621613										SC1	SC1^c^				2
MC	Wholesale market	Charleroi	50.390757	4.451212										SC1	SC1^c^				2
Total # PoE's monitored					8	12	4	2	23	13	13	13	3	23	23	21	7		

SC1 = scenario 1 (no establishment), SC1 INS = scenario 1 with IMS introduction followed by intensive survey (INS), SC2 = scenario 2 (locally established, < 25 km^2^), SC3 = scenario 3 (widely established, > 25 km^2^)

^a^Monitoring activities at EU in 2017 were part of the monitoring at and around RA

^b^Although the IMS is locally (scenario 2) or widely (scenario 3) established, a scenario 1 monitoring was implemented. At MM no spread was detected outside the 6-km buffer zone and control measures were implemented at the hotspot in 2019. At HA no spread was detected outside the used tyre import company in 2017 and in 2020 control measures were ongoing. SP belonged to the scenario 2 monitoring of HA

^c^Monitoring activities were stopped in June 2018

From 2007 to 2010 (MODIRISK project), 930 sampled sites were randomly chosen in urban, rural and natural areas based on the Corine Land Cover classes, while 45 sampled sites were specifically selected as potential points of entry of vectors [18 industrial sites (i.e. PoEs)] or pathogens (27 natural sites).

Since 2012, PoEs were selected yearly based on the qualitative risk assessment (QRA) of the ECDC guidelines [29]. The QRA was based on seven factors scoring the risk for introduction and establishment of IMS: import origin, import volume/frequency (compared per type of PoE), import method, import possibility at PoE, habitat suitability of the IMS around PoE, recent import of *Ae. albopictus* at PoE and evidence of import at this PoE type in other countries. Besides the QRA, the choice for inclusion of a specific PoE was also based on practical or financial considerations. Additionally, new high-risk PoEs were added, often replacing already monitored PoEs, after new information was obtained. For example, a new used tyre import company was identified in 2016 based on customs data collected through the HarmVect project [30]. In 2017, 11 of the 23 PoEs were pre-selected by the Belgian government based on the previous projects and expert advice. An overview of the selected PoEs per year can be found in Table 1.

### Sampling strategies

Since 2012, IMS surveillance in Belgium was implemented in line with the ECDC guidelines [29] with intensified surveillance mainly based on the monitoring strategy implemented in The Netherlands [31]. Sampling strategies differed according to the risk scenarios of IMS introduction and establishment [29] and adapted according to project needs (Additional file 1: Table S1). In scenario 1 (SC1—no presence of IMS) PoEs were surveyed to detect the introduction of IMS early. In case of a positive finding of IMS during a SC1 monitoring, surveillance was intensified to monitor the persistence of the IMS at the PoE and its spread into the surroundings (200–500 m buffer zone). In case of an IMS introduction at a parking lot, a SC1 monitoring was implemented at the next service station with a restaurant along the same highway. By targeting the next parking lot we aimed to assess the possible further spread along the highway. In most cases surveying a 200–500 m buffer zone around the parking lots was not considered an appropriate strategy because of the absence of a suitable environment for IMS. In scenario 2 (SC2), with IMS established locally covering an area of < 25 km^2^, the PoE and surroundings of the colonised area (in a 500 m–10 km buffer zone around the point of first detection at the PoE) were monitored to follow up the establishment and spread of the IMS. In scenario 3 (SC3), where IMS are widely established (covering an area of more than 25 km^2^), the seasonal abundance and spread of IMS were monitored at and around the colonised area, i.e. 6–8 km buffer zone around the point of first detection at the colonised area.

In general, the IMS monitoring was performed each year between April and November [except in 2012 (July–October), 2017 (August–November) and 2020 (August–October)]. This monitoring continued during winter months (1) at the lucky bamboo import company from 2016 onwards, because imported mosquitoes can reproduce indoors in the plant nursery during the winter, (2) once at the port of Antwerp (2013–2014) and (3) in case of scenario 3.

Adult mosquitoes were collected with Mosquito Magnet™ Liberty Plus/Executive/Independence traps (Woodstream Corp., Lititz, PA, USA), baited with octenol (since 2015) and CO_2_ (MMT), BG-Sentinel traps (Biogents, Germany), baited with BG-lure (BG), CDC gravid traps (Frommer Updraft, JW Hocke company, Gainesville, FL, USA) (GT) or BG-GAT traps (gravid *Aedes* traps, Biogents, Germany). The GT trap was mainly used to survey *Ae. japonicus* and *Ae. koreicus*. At used tyre and lucky bamboo import companies, BG and MMT traps were set up next to each other to increase attractiveness by using lures from both traps and CO_2_ from the MMT. Larvae were collected from potential larval habitats (PLHs). A PLH has been defined as a single vessel or a group of the same vessels (e.g. a stock of tyres, lucky bamboo containers in the same shelter) in which mosquito larvae can develop. Larval sampling (LS) was done mainly by netting with a fine-meshed aquarium net, but small PLHs were aspirated with a pipette or large syringe, or totally emptied into a white tray. Eggs were collected with oviposition traps (OT) consisting of a black plastic bowl (volume of 0.5 to 2 l) with drainage holes, filled for 2/3 with an infusion [oak (2012–2015) or hay (2016–2017)] or tap water (2018–2020) with a floating oviposition support [polystyrene piece (2012–2020) or wooden paddle (2020, for *Ae. japonicus*)].

The surveillance of IMS was coordinated and in large part completed by the Unit of Entomology of the Institute of Tropical Medicine, Belgium. To improve the cost-efficiency and sustainability of the monitoring, local partners assisted in trap handling in 2018, 2019 and 2020. The directorate of roads in Wallonia, primarily responsible for managing the road and highway network, and the Flemish Environment Agency agreed to voluntarily operate the OT at the parking lots and send the oviposition substrates to the Unit of Entomology. Also Belgian defence voluntarily operated BG and OT traps at the airport of Zaventem in 2018 and 2019.

### Mosquito identification

The adult specimens were killed by storing them at − 20 °C upon arrival at the laboratory. Larvae were killed in 80% ethanol in the field (2007–2016) or transported alive to the laboratory and killed by a thermal shock with hot water (70 °C) (2017–2020). After the thermal shock, the larvae were transferred in 80% ethanol and, after morphological identification, in the case of exotic specimens, in absolute ethanol. Morphological identification of adults and larvae was done with a stereomicroscope using dichotomic and digital keys [29, 32–37]. To confirm and validate the morphological identification of IMS and to identify damaged adults and larvae, DNA-barcoding was performed [18, 26, 27, 36, 38, 39].

The collected polystyrene pieces from OT were checked for the presence of IMS eggs in the laboratory by using a stereomicroscope. From 2007 to 2010 and from 2013 to 2019, a subsample of the eggs from the polystyrene piece (1–5 eggs per side) was always DNA-barcoded [18, 26, 27, 36, 40]. In 2012 and 2020 the positive polystyrene pieces or wooden paddles were immerged in water in secured containers, which were stored in a secured mosquito breeding room (2012 [25]) or climate controlled cupboard (2020). Hatched larvae (3rd or 4th instar) were stored in 80% (2012) or absolute (2020) ethanol for morphological identification. In case the eggs did not hatch, MALDI-TOF mass spectrometry [41] (2012 [24]) or DNA-barcoding (2020) was performed on the eggs for identification.

### Database management and analysis

From 2007 to 2010 a Personal Digital Assistant (PDA) and Global Positioning System (GPS) were used to enter data in the field [20], whereas in the other years mobile applications were used on a smartphone (VECMAP®, Avia-GIS, Belgium [42], in 2012 [24], as well as from 2017 to 2020, and Epicollect [43] from 2013 to 2016). All the PoE, trap and sampling information as well as the morphological identification results were stored in the data management system MS ACCESS (2007–2016) or VECMAP® (2017–2020) with a traceable and unique labelling system. Datasets from 2007 to 2020 are published on the Global Biodiversity Information Facility (GBIF) website [44–50].

A logistic regression (using R software [51]) was performed to investigate the trend in the percentage of positive PoEs for *Ae. albopictus* over the years. The time effect was introduced in the logistic regression as an independent variable. The outcome variable is the percentage of positive PoEs and the estimates of the logistic regression in this case correspond to the effect of 1-year increase in terms of log odds. Exponentiating these estimates gives the odds ratio. When the odds ratio is > 1 we consider there is an increase in finding a positive PoE in 1-year increase and a value < 1 means there is a decrease in the probability that a PoE is positive. The *P*-values allow us to conclude whether the results are significant by fixing the significance level at 5%.

## History and state of the art of invasive mosquito species in Belgium

Three IMS species were recorded in Belgium between 2000 and 2020: *Ae. albopictus*, *Ae. japonicus* and *Ae. koreicus*. *Aedes albopictus* is in the first stage of invasion ‘introduction’, *Ae. japonicus* in the second invasion stage ‘local establishment’ and *Ae. koreicus* in the third invasion stage ‘spread’. The invasion history differs among these species and will be discussed accordingly.

## *Aedes albopictus*

**Fig. 2:**
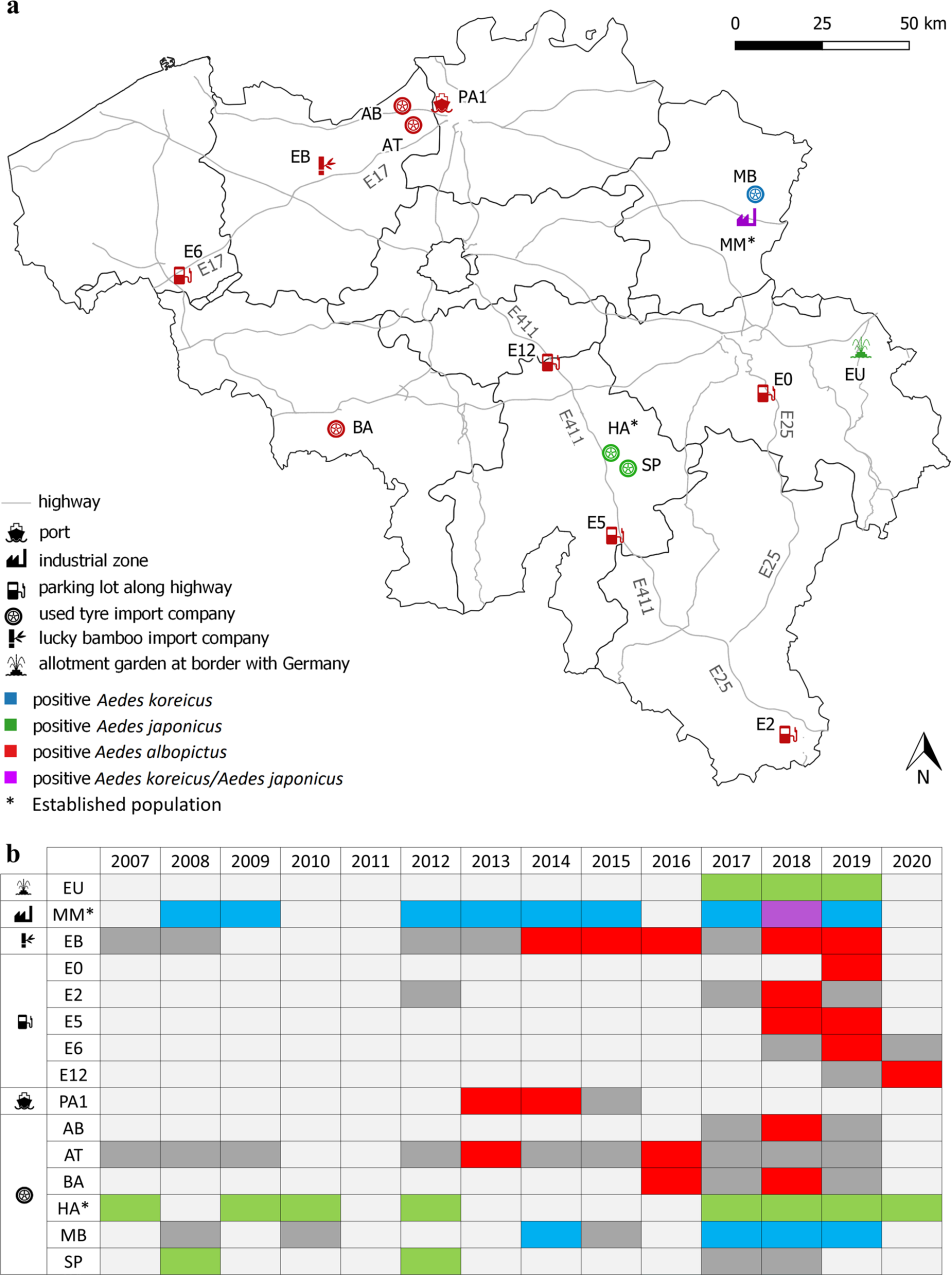
Map of Belgium with PoEs which were positive for invasive mosquito species (IMS) (the borders indicating the areas of Belgian provinces) (**a**). Indication of monitoring activities (not implemented: light grey), negative (dark grey) and positive findings per year of *Ae. albopictus*, *Ae. japonicus* and *Ae. koreicus* between 2007 and 2020 are tabled below for these PoEs (**b**)

*Aedes albopictus* was detected at ten PoEs spread throughout the Belgian territory in 2007–2020 (Fig. 2, Additional file 1: Table S2). Based on the observations made during this period, the species was not yet able to establish given that no evidence for overwintering was found. Yet, indoor and outdoor summer reproduction of *Ae. albopictus*, as indicated by detection of immature life stages, occurred at and around some PoEs where control measures were not implemented, or only implemented 2 to 3 months after the first detection of *Ae. albopictus* (Additional file 1: Table S2). As reproduction of *Ae. albopictus* in Belgium is possible, the time gap between the detection and control should be as small as possible to lower the risk of possible spread and establishment.

Typically, *Ae. albopictus* was collected between May and October. The earliest detection of *Ae. albopictus* (1 male and 2 females) during the mosquito season (April–November) in a non-sheltered environment was in May 2018 at the used tyre import company AB in Kallo. This finding precedes the earliest detection of the species in a non-sheltered environment in The Netherlands, applying similar methods and covering the same sampling period [52]. This early observation might be due to an early introduction combined with suitable climatic conditions in April–May 2018, which were exceptionally warm [53] and might have favoured the development and survival of the introduced individuals. The last specimens of *Ae. albopictus* were predominantly collected in September up to the end of October, comparable to the situation in The Netherlands [52]. Indoors, introduced *Ae. albopictus* were collected once as larva in February 2016, which was probably linked to the increased import of lucky bamboo from Guangdong (southern China) for the Chinese New Year. Furthermore, twice *Ae. albopictus* was collected in November (adults and larvae), again linked to the import of lucky bamboo. These findings point to the fact that the species can be introduced through the lucky bamboo trade at any time of the year [54]. The species was collected with OT, BG and MMT traps and by LS. All of these methods were suitable to detect *Ae. albopictus* at a first and last time point of a given year.

The companies trading used tyres and lucky bamboo plants are well-known introduction routes [52, 55, 56]. Since the first detection of *Ae. albopictus* in Belgium in 2000 [19], 12 *Ae. albopictus* introductions through used tyre and lucky bamboo trade were recorded (Additional file 1: Table S2). *Aedes albopictus* was detected at the used tyre import company AT in Vrasene in 2013 [26] and 2016. Furthermore, *Ae. albopictus* was detected in two other used tyre import companies: BA in Frameries (2016 and 2018) and AB in Kallo (2018). During the intensified surveillance, the species was also found in the 200–300-m perimeter around the point of first detection at BA in 2016 and at AB in 2018. In 2013 and in 2014, *Ae. albopictus* was intercepted from shipments with lucky bamboo plants at the port of Antwerp PA1, destinated for the lucky bamboo import company EB in Lochristi [26, 57]. Subsequently, the species was detected at this lucky bamboo company in 2014 and since then each year from 2015 to 2019, except in 2017. Indoor summer reproduction was found in 2014–2016, but stopped the following years after preventive larviciding was implemented. Furthermore, 32 *Ae. albopictus* eggs were collected in one ovitrap just outside the plant nursery in 2015, indicating potential reproduction outside.

Control measures [mainly larviciding with Bti (*Bacillus thuringiensis israelensis*) and Bs (*Bacillus sphaericus*), granular and liquid formulations] were implemented since 2014 on an ad hoc basis with an evolution from reactive to preventive measures in Flanders. When preventive larviciding was implemented at these well-known and high-risk PoEs, few (lucky bamboo) or no (used tyre) new detections were made anymore. Other preventive measures such as obligate covering of used tyres and obligate treatment of lucky bamboo water should be taken to regulate the used tyre and lucky bamboo import in Belgium. In The Netherlands such a regulation for prevention of *Ae. albopictus* is already in place in the form of covenants and legislation for import of these commodities (used tyres and lucky bamboo) [52]. However, even with these regulations in place, active surveillance remains necessary to detect introduction of IMSearly and evaluate control measures [52].

The detection of *Ae. albopictus* at Belgian parking lots since 2018 (Fig. 2, Additional file 1: Table S2) points towards a short-distance introduction pathway, already observed in other European countries [55, 58–62] but new for Belgium. For example, ongoing introductions of *Ae. albopictus* in southern England occurred via ground vehicular traffic through train and ferry from nearby established populations in France [63]. The expanding population of *Ae. albopictus* in northern France (departments of Val-de-Marne, Seine-Saint-Denis, Hauts-de-Seine, Seine-et-Marne, Essonne, Aisne Bas-Rhin) or in Germany (Baden-Württemberg and Hesse) [64–66] most likely is the source of the introduced *Ae. albopictus* found at the parking lots in Belgium, as these are located within a 2–2.5-h drive from these populations [29]. For vehicles coming from colonised areas in northern France and Germany, the monitored parking lots were often the first stop after crossing the border. Also the recent detections at parking lots in The Netherlands at the northern Belgian border [67] suggest that *Ae. albopictus* can be introduced anywhere in Belgium. Certainly, *Ae. albopictus* populations are progressively establishing closer to Belgium and more frequent short-distant introductions will take place in the coming years.

### *Aedes japonicus*

*Aedes japonicus* was detected at four PoEs in the period 2007–2020 and is locally established at one PoE (Fig. 2, Additional file 1: Table S3). The species was typically collected between May and October, but larvae could be collected as early as March, while adults were sampled until November. *Aedes japonicus* was collected with OT, GT, BG and MMT traps and by LS. All these methods were suitable as last detection method for *Ae. japonicus*. MMT, OT, GT and LS methods were also suitable as first detection method for *Ae. japonicus* in a given year, although LS was the most frequent first detection method for this species.

Since 2002, *Ae. japonicus* has been locally established at a used tyre import company in Natoye (HA) [18]. At that time tyres were imported from their home range in Japan and from their invasive range in the USA, where the species is established, making the international used tyre trade the probable introduction pathway [18]. For > 10 years, *Ae. japonicus* was reported as locally established in a 2-km perimeter around the tyre import company HA (Additional file 1: Table S3). The species was intercepted twice, in 2008 and 2012, at the used tyre import company SP, which is located 2 km southeast of HA. No further spread of *Ae. japonicus* was detected in 2012. This was in strong contrast to the fast spread of the species observed in other European countries [68]. In 2012, the first vector control measures (removal of PLH and larviciding with Bti and Bs, granular and liquid formulations) were implemented at both companies and in the surroundings, which drastically reduced the population size of *Ae. japonicus*, but the species was still present and detected up to 2 km mainly in the southwestern direction [24]. Subsequently a large vector control programme was executed between 2013 and 2015 after which the *Ae. japonicus* population was considered eliminated [69–71]. This was the first Belgian IMS elimination campaign ever. In 2017, the monitoring of *Ae. japonicus* focused on confirming the elimination at and around the used tyre import company HA. *Aedes japonicus* was rediscovered at the same PoE HA in 2017, and this was reconfirmed in 2018. In 2019 the population density at the tyre company HA increased strongly compared to 2017 and 2018 (Additional file 1: Table S3). The percentage of positive oviposition substrates from OT increased from 12% in 2017 to 13% in 2018 and 32% in 2019 (the sampling periods and efforts were comparable in 2017–2019). The detection of *Ae. japonicus* eggs and larvae up to 750 m from HA (forest ‘Bois Henrard’, private garden in Vincon, Ciney) in 2019 implies that *Ae. japonicus* spread again in southwest direction as it was the case in 2012. It seems that the small forest located southwest of the tyre company forms a good ‘green corridor’ for the spread of the mosquito [40, 72]. At the northeast side, the company is surrounded by open meadows, which possibly hamper the spread of the population in that direction.

An investigation of the genetic variation at seven microsatellite loci indicated that remnants of the original population were still present, although no detections were done in 2015 and 2016 during the control campaign [70]. Natural or cryptic larval habitats (tree holes, plastic sheet covering wood, plastic waste) in the small forest probably played a role as ‘refuge’ [22, 40, 73]. The current admixed population displayed a higher allelic richness than the original population, which points towards at least one re-introduction of *Ae. japonicus* from an external source population after the elimination campaign [40]. Tyres are regularly imported from an *Ae. japonicus*-colonised area in Germany [74], which might be the possible source of this new introduction. Currently, the tyre company only imports tyres from Europe (mainly Luxembourg, France, Italy, Spain, Germany and The Netherlands) (HA, personal communication). German *Ae. japonicus* populations display high invasiveness potential as indicated by their fast spread after establishment all over the country [75]. High genetic diversity might be a component of their invasiveness success [75, 76]. A new introduction possibly increased the genetic diversity of the Belgian population, which could explain the apparently faster spread seen in 2019 compared to that before 2012. In 2020 control measures (removal of PLH and larviciding with Bti, granular formulation) were implemented at HA and in the 500-m buffer zone (forest ‘Bois Henrard’, areas in the hamlet Vincon). During the treatment of larval habitats in 2020, immature life stages were still detected at the premises of the used tyre import company HA. Controlling *Ae. japonicus* at HA has proven to be challenging and given the continuous tyre trade with colonised countries like Germany, new introductions are likely to occur, with further genetic admixture.

In The Netherlands, *Ae. japonicus* was associated with allotment gardens [77, 78]. Likewise, this species was found in an allotment garden along the country border with Germany (EU) from 2017 to 2019 (Additional file 1: Table S3). The monitoring results, supported by a genetic investigation [40], point to the phenomenon of multiple introductions in Belgium from the nearby population in West Germany [64, 68, 79]. In fact, the late detection in June 2018 and the limited number of specimens collected, together with the absence of adults or of egg-laying females (i.e. no eggs were collected with OT) in 2019 suggest the absence of an established population in the allotment garden. Yet, the detections of eggs and larvae of *Ae. japonicus* in the 200-m perimeter around the allotment garden later in the season in 2018 and 2019 confirmed summer reproduction of the species. The range expansion of *Ae. japonicus* in Austria, Italy and Switzerland seems to be mainly driven by active dispersion next to passive ground transport [62, 80–82]. Whether the introductions in Belgium occurred via one or the other pathway could not be determined from the genetic dataset [40].

At the industrial area in Maasmechelen (MM) close to the German border, *Ae. japonicus* adults were found only once between mid-June and mid-July 2018 (Additional file 1: Table S3), and thus a single introduction is hypothesised, which is supported by the genetic results [40]. Interestingly, it was the first time that *Ae. japonicus* and *Ae. koreicus* co-occurred at a PoE in Belgium. Co-occurrences of *Ae. japonicus* and *Ae. koreicus* have been previously reported from Germany, Switzerland, Slovenia and Italy [37, 82].

### *Aedes koreicus*

*Aedes koreicus* was detected at two PoEs in 2007–2020 and is widely established at one PoE (Fig. 2, Additional file 1: Table S4). Adults of *Ae. koreicus* were caught from May to the beginning of October with a peak in August and September in 2009 and in June and July in 2018, and larvae were found from March until October. The traps OT, GT, BG, BG-GAT and MMT as well as LS were suitable methods to collect *Ae. koreicus*, whereas MMT, LS and OT were appropriate for first detection in a given year. Similarly to *Ae. japonicus*, the most frequent first detection method for *Ae. koreicus* was LS. The methods MMT, LS and GT were suitable as last detection method for *Ae. koreicus*.

Since *Ae. koreicus* was first found in 2008 in the forest patch next to the industrial area 'Op de Berg' (MM) [21], the species has established at the industrial area (Additional file 1: Table S4). Although the import route of this IMS into Belgium remains unknown, international trade was speculated because of the large industrial zone surrounding the area [21]. Similar to the other invasive species, *Ae. koreicus*, uses a variety of human-made breeding sites [37, 83]. In Belgium these included metal containers [such as old construction equipment (mainly excavator heads)], small and large tyres, plastic containers (such as buckets, trays) and plastic sheets [27]. *Aedes koreicus* was also found once in temporary muddy road tracks (2008) [21]. In 2009 and 2017, larvae and eggs were collected up to 4 km to the east of MM. The detection of some larvae in 2014 and adults in 2017–2019 at the used tyre import company in Dilsen-Stokkem (MB) at 5.4 km from the industrial area MM was the furthest detection of this species. These frequent observations at > 2.8 km from the point of first detection indicate a widespread *Ae. koreicus* population. However, most specimens can be found at the industrial area MM itself, which remains the hot spot for this species. At MM the locally established population was reduced in 2019 after the first control campaign (larviciding with Bti, granular formulation) in the same year (Additional file 1: Table S4). The fact that mainly adults were collected at the used tyre company MB, often late in the reproduction season (September–October), suggests seasonal spread from the established population at MM. However, introduction by tyre trade at MB cannot be ruled out since the company imports tyres from many countries (MB, personal communication). After activation of the monitoring plan for a SC3 in 2018, no specimens were collected in the 6–8-km buffer zone around MM. It remains unclear why the species did not disperse faster, in contrast to other populations in Europe where it also spreads passively by road transport [37, 62, 83]. The spread found in the northeast of the industrial area might be explained by the presence of the forest ‘Mechelse Bos’, which forms a good ‘green corridor’ for the mosquito to spread compared with the open terrain of the sand quarry and the heath at the other sides of the industrial area. In general, mosquitoes prefer to fly through bushes and shrubs and avoid dry and open terrain [29]. However, low availability of PLH, other than tree holes, in the forest ‘Mechelse Bos’ next to the industrial area MM might have slowed down the active spread. We recommend to further investigate the host preferences of the Belgian *Ae. koreicus* population, as there have never been biting complaints from the people working and living at and around the industrial area [21]. Hypothetically, the population could feed mainly on non-human mammals, in contrast to other studies [84], which might explain the apparent lack of passive spread through ground traffic.

## Implications for public health and control

Based on the current spread of *Ae. albopictus*, particularly in France and Germany, on the increasing number of interceptions of this species in Belgium and on the suitability models developed in Europe [6, 85, 86], establishment of this species in Belgium is to be expected. Not only *Ae. albopictus*, but also *Ae. japonicus* is gaining more territory in neighbouring countries: Germany [58, 61, 64, 87], The Netherlands [67], Luxembourg [88], France [60, 66] and the UK [63, 89]. Anno 2020, both species have widespread established populations less than 200 km from Belgium, and *Ae. japonicus* is even reaching the Belgian border in the east. European *Ae. albopictus* and *Ae. japonicus* are able to transmit DENV, CHIKV and ZIKV in the field or in the laboratory, respectively [1]. Local transmission events of these arboviruses occur in Europe, primarily in Mediterranean countries [11–13]. As international movement of people and the number of dengue cases are both increasing worldwide [4, 90, 91], the number of travellers potentially infected by these exotic pathogens entering into Europe are also expected to increase. The number of imported CHIKV and DENV infections in Europe are related to the epidemiological situation of endemic disease in regions where the viruses circulate [92, 93]. In some years a dramatic increase of imported cases is linked to outbreaks in endemic countries [10, 94–96]. In combination with a possible future widespread establishment of *Ae. albopictus* in Belgium, the arbovirus transmission risk will increase.

We observed a clear increasing trend of the percentage of positive PoEs for *Ae. albopictus* in Belgium over the last 13 years (logistic regression coefficient: 0.517; *P* < 0.00001) with parking lots contributing most to the increased number of PoEs since 2018. The corresponding odds ratio is 1.67; this means there is 67% significant increase in the probability of PoEs being positive with 1-year increase. On top of the still important long-distance IMS import with well-defined PoEs (especially the used tyre and lucky bamboo import companies), now also short-distance import with less-defined PoEs occurs. For Belgium, the passive ground transport is a new and important introduction pathway for especially *Ae. albopictus* and probably also for *Ae. japonicus*. Natural dispersal for the latter species into Belgium is expected as well. Belgium literally is at the front of their invasion range and highlights the need for an integrated management programme in contrast to the current short-term project-based monitoring. Such a programme should include not only active and passive surveillance for the detection of IMS at both well-known and unknown PoEs, but also a clear and complete control management plan at national and regional levels setting out clear criteria for action, control methods and strategies with appropriate implementation, supervision and evaluation. The decision and implementation of control of IMS species in Belgium differ between regions and is not always based on epidemiological and entomological risk scenarios such as described by Roiz et al. [97]. The IMS control in Belgium is often too ad hoc as it is done reactively or proactively depending on the available biocides, budget and sometimes political priorities, and it also depends on what the policy is in neighbouring countries, e.g. the lack of *Ae. japonicus* management in Germany. Finally, involvement of local authorities (municipalities, provinces and local Public Health units), social mobilisation, cross-border collaboration and a link with Public Health surveillance (including travel epidemiology) should be added to the surveillance and control management plan.

## Conclusion

Since the first IMS detection in Belgium in 2000, repeated introductions have occurred between 2007 and 2020 at a total of 14 PoEs. *Aedes albopictus* currently enters Belgium through three pathways: lucky bamboo trade, used tyre trade and passive ground transport. *Aedes japonicus* enters through the used tyre trade and probably passive ground transport, while it is unclear how *Ae. koreicus* was initially introduced. The IMS *Ae. japonicus* and *Ae. koreicus* have established without spreading far in Belgium.

The IMS import through passive ground transport is new for Belgium and creates a situation with both well-known PoEs and less-defined PoEs. The control management actions at well-known PoEs with long-distance introductions are more straightforward than at less-defined PoEs with short-distance introductions. This will be a new challenge in the coming years for Belgium as established populations of *Ae. albopictus* and *Ae. japonicus* are approaching the country border and introductions through passive ground transport are expected to become more frequent. *Aedes albopictus* is expected to become established in Belgium in the coming years, hence increasing the likelihood of local arbovirus transmission. The implementation of a sustainable, structured and long-term IMS management programme in Belgium, integrating active and passive entomological surveillance, vector control and Public Health surveillance, is therefore pivotal.

## Supplementary Information

**Additional file 1:** **Table S1.** Overview of the trapping methods used to monitor invasive mosquito species (IMS) in Belgium during the different years (and projects) and in different risk scenarios, indicating the number of traps or larval sampling visits per site and the frequency of trapping or larval sampling. **Table S2.** *Aedes albopictus* detections in Belgium between 2007 and 2020 at the ten points of entry (PoEs) per year including the sampling perimeter, collection and detection methods, collection (light grey) and detection (dark grey) period, control measures (X), number of individuals (total, females, males, larvae and eggs) and project.** Table S3.** *Aedes japonicus* detections in Belgium between 2007 and 2020 at the four points of entry (PoEs) per year including the sampling perimeter, collection and detection methods, collection (light grey) and detection (dark grey) period, the control measures (X), number of individuals (total, females, males, larvae and eggs) and project.** Table S4.** *Aedes koreicus* detections in Belgium between 2007 and 2020 at the two points of entry (PoEs) per year including the sampling perimeter, collection and detection methods, collection (light grey) and detection (dark grey) period, control measures (X), number of individuals (total, females, males, larvae and eggs) and project.

## Data Availability

Data supporting the conclusions of this article are included within the article and its additional file. The datasets generated and analysed during the current study are available in the GBIF repository [44–50].

## Abbreviations

AB: Used tyre import company in Kallo
AT: Used tyre import company in Vrasene
BA: Used tyre import company in Frameries
BG: BG-Sentinel trap
BG-GAT: BG gravid *Aedes* trap
Bti: *Bacillus thuringiensis israelensis*
CHIKV: Chikungunya virus
DENV: Dengue virus
DiMoC: Diversity components in Mosquito-borne diseases in face of Climate change
DNA: Deoxyribonucleic acid
E0: Parking lot Sprimont (Noidré)
E12: Parking lot Eghezée (Aische-en-Refail)
E2: Parking lot Messancy (Hondelange)
E5: Parking lot Houyet (Wanlin)
E6: Parking lot Kortrijk (Marke)
EB: Lucky bamboo import company in Lochisti
ECDC: European Centre for DiseasePrevention and Control
EU: Allotment garden in Eupen
EXOSURV: Implementation of SURVeillance of EXOtic mosquitoes in Belgium
FASFC: Federal Agency for the Safety of the Food Chain
GPS: Global Positioning System
GT: Gravid trap
HA: Used tyre import company in Natoye
IMS: Invasive mosquito species
LS: Larval sampling
MALDI-TOF: Matrix-assisted laser desorption-ionization-time of flight
MB: Used tyre import company in Dilsen-Stokkem
MEMO: Monitoring of Exotic MOsquitoes in Belgium
MM: Industrial area in Maasmechelen
MMT: Mosquito Magnet™ trap
MODIRISK: MOsquito vectors of Disease: spatial biodiversity, drivers of change, and RISK
OT: Oviposition trap
PA1: Port of Antwerp (right bank)
PDA: Personal digital assistant
PLH: Potential larval habitat
PoE: Point of entry
QRA: Qualitative risk assessment
SC1: Scenario 1
SC2: Scenario 2
SC3: Scenario 3
UK: United Kingdom
WHO: World Health Organisation
ZIKV: Zika virus
